# A Simple, Rapid and Reliable Protocol for Extraction of High Quality Bacterial Genomic DNA Directly from Potato Tubers for Efficient PCR-Based Surveillance and Molecular Characterization of *Ralstonia solanacearum*

**DOI:** 10.3390/mps9030084

**Published:** 2026-05-31

**Authors:** Brian Mwangi, Joshua M. Njiru, Sarah A. Wandili, Kennedy K. Gachoka, Kenneth Mburu, Geoffrey Muriira, Henry Rotich, Elvince Ager, Evans N. Nyaboga

**Affiliations:** 1 Department of Biochemistry, University of Nairobi, Nairobi P.O. Box 30197-00100, Kenya; bmwangi918@gmail.com (B.M.); agerelvince@gmail.com (E.A.); 2 Department of Biological Sciences, Meru University of Science & Technology, Meru P.O. Box 972-60200, Kenya; swandili@must.ac.ke (S.A.W.); kgachoka@must.ac.ke (K.K.G.); 3 Research and Development, Kenya Bureau of Standards, Nairobi P.O. Box 54974-00200, Kenya; muriirag@kebs.org (G.M.); rotichh@kebs.org (H.R.); 4 Department of Life Sciences, South Eastern Kenya University, Kitui P.O. Box 170-90200, Kenya; kmburu@seku.ac.ke

**Keywords:** bacterial genomic DNA extraction, DNA quality, molecular diagnostics, *Ralstonia solanacearum*, sanger sequencing

## Abstract

Potato (*Solanum tuberosum* L.) is an important staple and food security crop to many communities in the world. However, potato production and quality is greatly constrained by bacterial wilt, a disease caused by a soil-borne pathogen, *Ralstonia solanacearum*. *Ralstonia solanacearum* can be managed through clean seed systems and therefore laboratory testing is a pre-requisite for seed certification to confirm the absence of the pathogen in potato seeds before planting. Molecular diagnostics is the gold standard for detection of *R. solanacearum* in potato seeds. However, the extraction of genomic DNA from *R. solanacearum* for molecular diagnostics is complex, tedious, lengthy and/or costly procedure. A simple, rapid and reliable DNA extraction protocol is required for use in routine molecular diagnosis of *R. solanacearum*, a high-risk quarantine pathogen. In this study, we developed a simple and rapid protocol for extracting genomic DNA from symptomatic and asymptomatic potato tubers infected with *R. solanacearum* and verified its efficiency for the detection and molecular characterization of the pathogen. The protocol was developed from the evaluation of distilled water, Tris-EDTA (TE) and Tris buffer as a base solution for tissue maceration. The DNA quantity and integrity was determined using the NanoDrop 2000C spectrophotometer and agarose gel electrophoresis, respectively. Both hot and cold solutions produced intact high molecular weight genomic DNA of sufficient yield and purity for molecular-based applications. The detection and determination of phylotypes of *R. solanacearum*, based on conventional and multiplex polymerase chain reaction (PCR), amplified the expected 280 and 372 bp amplicons, respectively, confirming that the quantity and quality of the extracted pathogen genomic DNA was sufficient for molecular diagnostic applications. The sequencing of the amplified products of the *endoglucanase* gene produced good quality sequences, which confirmed the *R. solanacearum* isolates to be members of phylotype II sequevar 1. This protocol is a simple, fast and reliable tool for the extraction of sufficient genomic DNA with high quality, directly from *R. solancearum*-infected potato tubers for PCR and sequencing applications. Its simplicity and throughput make it valuable for use in routine diagnostics and can be adopted by certification programs to ensure distribution of clean potato seeds to farmers.

## 1. Introduction

Potato (*Solanum tuberosum* L.) is the third-most important from crops globally after maize, wheat and rice and ranked first among vegetable crops [1,2,3]. It is widely cultivated and consumed as a primary food crop for over 1.3 billion people [4]. However, bacterial wilt caused by *Ralstonia solanacearum* species complex (RSSC) is a significant threat to potato production and quality, leading to huge economic losses [5,6,7]. *Ralstonia solanacearum* has been reported to infect more than 1.5 million hectares of fields for potato production globally and has a broad host range comprising 450 plant species, with the family Solanaceae being highly susceptible [8]. It is the most destructive bacterial pathogen due to its wide host range, adaptability, high genetic diversity and huge economic losses [9]. As a result, *R. solanacearum* has been placed under the quarantine list of pathogens with strict control over the entry of potato seeds and tubers into many countries. However, studies have demonstrated that there is a spread of RSSC between international borders and this is mainly through infected potato tubers used as seeds [6].

The use of clean and healthy potato seeds is the most effective strategy to prevent the spread and dissemination of *R. solanacearum* to disease-free locations. The potato seed system is largely informal in many developing countries contributing to the spread of *R. solanacearum* through latently infected tubers [10]. This is a significant problem in potato production globally [11]. For example in Kenya, the formal potato seed system contributes less than 3% of the seed demand nationally [12,13]. Furthermore, there are no restrictions implemented in regional and cross-border movement of potato seed. The informal seed systems and unrestricted seed movement within developing countries and cross-border poses a significant threat in dissemination of *R. solanacearum* to regions that are not infected and may also introduce new genetic variants. This warrants the need for a robust molecular diagnostic system to facilitate the implementation of point-of-care molecular detection and allow higher sample throughput for the case of quarantine pathogens such as *R. solanacearum*.

Diagnostic methods based on nucleic acid amplifications have been developed and accelerated the detection and identification of the phylotypes of RSSC [14,15,16]. However, the first and most important step for efficient use of nucleic acid amplifications is the extraction of pathogen genomic DNA. The current methods for DNA extraction require many steps which includes bacterial (pathogen) isolation from host plants, pathogen culture in different types of broth, and enrichment media as a DNA pre-extraction preparation step. The enrichment of *R. solanacearum* requires at least 72 h and some compounds in the selective media have been reported to inhibit PCR [17]. In addition, the enrichment media for culturing *R. solanacearum* such as semi-selective medium—South Africa (SMSA) and triphenytetrazolium chloride (TZC)—are expensive. The enrichment of RSSC by culture is not required in PCR-based diagnostics and this is an advantage because of the reduced analytical turn-around times.

Several protocols have been developed and adopted in many laboratories for extraction of genomic DNA from plant bacterial pathogens including RSSC [5,18,19]. Although these procedures are well established and guarantee high quality DNA for various molecular applications, most protocols are complex and rely on expensive systematic procedures with toxic and hazardous organic chemicals and reagents such as phenol, isoamyl alcohol and chloroform. In addition, the protocols are time-consuming due to the several purification steps needed to remove other cell components, inhibitors or degrading enzymes before the DNA can be subjected to molecular analysis. The high risk of contamination related to the steps for the isolation of DNA and the use of toxic and hazardous chemicals make these procedures difficult for processing a large sample size and implement them in a routine laboratory. Many commercial kits for extraction of bacterial genomic DNA directly from infected plant tissues are available but they are expensive and, most times, require optimization [20,21]. The aforementioned disadvantages in sample preparation protocols for extraction of *R. solanacearum* genomic DNA impedes the implementation of rapid molecular diagnostics in resource-limited settings especially in developing countries. For these reasons, there is need for a simple, rapid, safe and reliable protocol for genomic DNA extraction to replace the costly and more time-consuming bacteria culture methods.

To overcome the limitations of the previous methods (e.g., labor-intensive, time-consuming, high cost, hazardous and highly toxic chemicals), the current study was performed to develop a simple, rapid, safe and inexpensive protocol suitable for pathogen genomic DNA extraction for molecular-based diagnostics. The protocol eliminates the utilization of toxic and hazardous organic reagents and chemicals, because the entire procedures were conducted in distilled water, TE or Tris buffer, which are directly used as components in PCRs. The present study aimed to develop a simple, rapid and safe protocol for extraction of bacterial genomic DNA directly from potato tubers infected with *R. solanacearum* for efficient molecular detection and characterization of the pathogen. The developed DNA extraction protocol could be used for routine diagnostics and can be adopted by certification programs to ensure distribution of clean potato seeds to farmers.

## 2. Experimental Design

### 2.1. Sampling of Potato Tubers and Preparation of Samples

**Figure 1:**
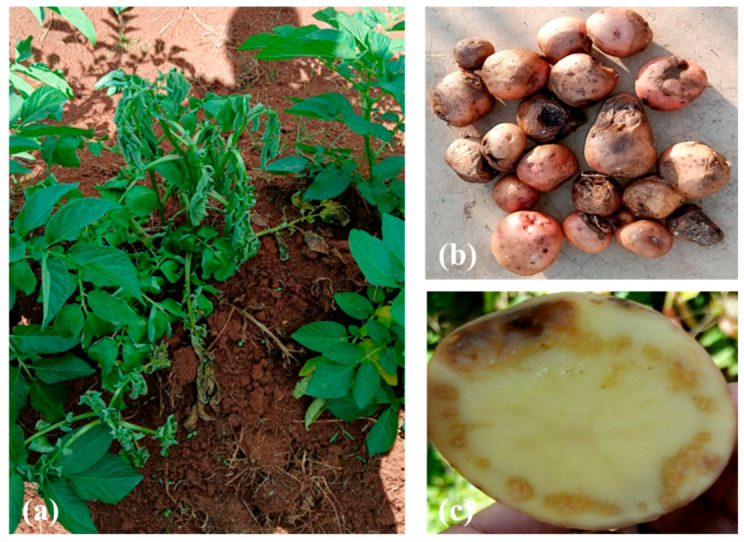
Symptoms of bacterial wilt (caused by *Ralstonia solanacearum*) in potato plants in the field (**a**), and tubers after harvest (**b**,**c**). Infected potato tubers from field plants with and without symptoms were used as samples for analysis.

Samples were sourced from major potato growing regions namely Nyandarua, Meru and Uasin Gishu Counties in Kenya. Potato tubers were harvested from plants exhibiting bacterial wilt symptoms (Figure 1), placed sample collection bags under cool conditions and transferred to the laboratory for analysis. The potato tuber samples were processed as described by Abdurahman et al. [22]. Potato tubers of healthy plants established in the greenhouse at the University of Nairobi were used as negative controls for the experiments. The tubers were washed using running tap water and then submerged in 70% ethanol for 2 min for surface sterilization. The tubers were then submerged in 1% NaOCl (sodium hypochlorite) for 60 s and then rinsed five times with sterile double distilled water. The stolon of each of the tubers was aseptically cut to expose the interior of the tuber. The samples that displayed vascular browning were utilized for extraction of pathogen genomic DNA for further analysis. Genomic DNA was extracted from pure cultures of *R. solanacearum* isolated from sample EC3, using the previously described method [6] as a positive control for the study. Three independent biological replicates were used for each of the solution used for DNA extraction.

### 2.2. Detection of Ralstonia solanacearum Based on PCR Amplification

The PCR amplification of a 282 bp target region of the 16S rRNA gene was based on 759/760 primer pair (Table 1) [8,22]. PCR amplifications were carried in volumes of 25 µL comprising 12.5 µL of 2× Taq polymerase master mix (Ampliqon), 0.5 µL of each of forward and reverse primer (10 mM), 2 µL of extracted DNA template and 9.5 µL of nuclease-free water. The PCR thermocycling conditions included initial denaturation for 4 min at 95 °C, 35 cycles of denaturation (40 s at 95 °C), annealing (40 s at 55 °C) and extension (30 s at 72 °C) followed by final extension (5 min at 72 °C). The amplicons were visualized against a 1 kb ladder (Fischer Thermo Scientific, Waltham, MA, USA) using 1.5% (*v*/*v*) agarose gel electrophoresis in 1× TAE buffer stained with ethidium bromide. The gel was run at 100 volts, 3 amperes and 300 watts for 45 min and the amplicon bands were visualized using UV transilluminator (BioRad, GMbh—FeldKirchen, Germany).

### 2.3. Multiplex PCR for Identification of Ralstonia solanacearum Phylotypes

Multiplex PCR was used to identify *R. solanacearum* phylotypes based on four forward primers and one unique conserved reverse primer (Table 1) [14]. The total reaction volume of each sample in multiplex PCR was 25 µL containing 12.5 µL of 2× Taq polymerase master mix (Ampliqon), 0.6 µL of forward primer (10 mM), 2.4 µL of reverse primer (10 mM), 2.5 µL of extracted DNA and 5.2 µL of nuclease-free water. The PCR thermal cycling conditions included initial denaturation for 5 min at 96 °C, 35 cycles of denaturation (15 s at 95 °C), annealing (30 s at 55 °C) and extension (30 s at 72 °C) followed by final extension (10 min at 72 °C). The amplicon bands were visualized as described in the PCR amplification for detection of *R. solanacearum*. Multiplex PCR was carried out on DNA extracted from asymptomatic samples using sterile distilled water to confirm the applicability of the method for extraction of *R. solanacearum* DNA in order to demonstrate the performance of the protocol in low titer or latent infection.

### 2.4. PCR Amplification and Sequencing of PCR Products for Identification of Sequevars

Phylotypes of RSSC were classified into distinct sequevars based on nucleotide differences in the *egl* gene (partial endoglucanase gene) [24]. The sequevars were identified by PCR amplification of *egl* gene using Endo-F and Endo-R primer pair (Table 1), which targeted the 750 bp region of the gene. Two samples were selected from each region and DNA was isolated using cold sterile distilled water. The PCRs were conducted in volumes of 50 µL comprising 25 µL of 2× Taq polymerase master mix (Ampliqon, Odense, Denmark), 1 µL of each forward and reverse primer (10 mM), 3 µL of extracted DNA and 20 µL of nuclease-free water. The PCR thermal cycling conditions included 4 min at 95 °C, 35 cycles of initial denaturation (40 s at 95 °C), annealing (40 s at 70 °C) and extension (30 s at 72 °C). A final extension of 5 min at 72 °C was used. The bands of the amplicons were visualized as described in the PCR amplification for detection of *R. solanacearum*. The confirmed bands of the PCR products were then purified using QIAquick PCR purification kit and purified samples sent to Macrogen, Europe for bidirectional Sanger sequencing.

### 2.5. Nucleotide Sequence Analysis and Phylogenetic Inference

The resulting chromatograms obtained from the sequencing were transferred to the software BioEdit for visualization, cleaning and construction of contiguous sequences (contig sequences). The resultant contig sequences were used for BLASTn search in NCBI database and the relevant sequences that spanned different sequevars were selected. The resultant sequences mined from GenBank were transferred to the phylogeny service platform NGphylogeny.fr where MSA (multiple sequence alignment) was conducted using the MUSCLE algorithm and curated by the BMGE algorithm [25,26]. The curated MSA was then used to determine the best substitution model by employing SMS [27]. This model was then used to infer phylogeny using the maximum likelihood method implemented using PhyML 3.0 [28]. The resultant tree was transferred to FigTree for visualization.

### 2.6. Statistical Analysis

The statistical analysis was performed between the different treatments using GraphPad prism software (version 10.4.1). Concentration and purity of the extracted DNA from hot and cold treatments were compared and *t*-test was performed.

### 2.7. Materials and Equipment

#### 2.7.1. Materials

- Ethylenediaminetetraacetic acid disodium salt, EDTA Na_2_ (Sigma Aldrich (St. Louis, MI, USA), cat. no. 102075972)
- Tris base (Merck (Darmstadt, Germany), cat. No. 77-86-1)
- Ethidium bromide solution (Sigma Aldrich, cat. no. E1510)
- 1 kb ladder (Fischer Thermo Scientific, Waltham, MA, USA)
- Nuclease-free water (Invitrogen (Carlsbad, CA, USA), cat. no. AM9920)
- Agarose molecular biology grade (BioRad (Singapore), cat. no. 1613101)
- 0.5–10 μL disposable tips (ULPlast, OM-10-RF-C, Warszawa, Poland)
- 1–200 μL disposable tips (ULPlast, OM-200-RF-Y, Warszawa, Poland)
- 100–1000 μL disposable tips (ULPlast, OM-1000-RF-B, Warszawa, Poland)
- Microcentrifuge tubes, 1.5 mL and 2 mL (Eppendorf, Hamburg, Germany)

##### Stock Solutions

- Tris-HCl (1 M, pH 8.0): To prepare it, dissolve 6.55 g of Tris base in 400 mL distilled water. Use concentrated HCl to adjust the pH. Adjust the final volume with distilled water to 0.5 L.
- EDTA (0.5 M): To prepare it, dissolve 93.05 g in 400 mL distilled water. Mix thoroughly using a magnetic stirrer, use NaOH to adjust pH to 8.0 and then adjust final volume 0.5 L using distilled water.

##### Buffer Solutions

- Tris buffer: 10 mM Tris-HCl (pH 8.0) (autoclaved). Mix 5 mL of 1 M Tris-HCl (pH 8.0) and adjust the volume to 0.5 L with deionized water. Autoclave and store at room temperature at 23 ± 2 °C for ≤12 months.
- Tris-EDTA (TE) buffer: This consists of 10 mM Tris-HCl (pH 8.0) and 1 mM EDTA (pH 8.0) and autoclaved. To prepare it, mix 5 mL of 1 M Tris-HCl (pH 8.0) and 1 mL of 0.5 M EDTA (pH 8.0) and adjust the volume to 0.5 L with deionized water. Autoclave and store at room temperature at 23 ± 2 °C for ≤12 months.

### 2.8. Equipment

- Micropipettes, p10 µL, p20 µL, p50 µL, p200 µL and p1000 µL
- 1.5 mL and 2.0 mL microcentrifuge racks
- Nanodrop 2000C spectrophotometer (Thermo Fisher Scientific, Wilmington, DE, USA)
- Veriti thermocycler (Bio-Rad, Singapore)
- Gel electrophoresis chamber (Bio-Rad, Hercules, CA, USA)
- pH Meter (LAQUA F-71; Horiba, Kyoto, Japan)
- Tabletop centrifuge (Eppendorf 5702, Eppendorf, Hamburg, Germany; Cat. No. 5702000062)
- UV transilluminator (BioRad, GMbh—FeldKirchen, Germany)

## 3. Procedure for Extraction of Bacterial Genomic DNA

Three different solutions, i.e., sterile distilled water, TE buffer, and Tris buffer were used for extraction of genomic DNA for each of the potato tubers infected with *R. solanacearum*. Both cold (at room temperature at 23 ± 2 °C) and boiled solutions were used. Three independent biological replicates were used for each of the treatments (solutions) used for DNA extraction.

1. Boil sterile distilled water, TE buffer and Tris buffer in water bath at 98 °C for 10 min.
2. Cut small rectangular cores (measuring 3 × 5 mm) containing vascular tissues (approximately 2 g) for the stolon end of each potato tuber.
3. Place the rectangular cores in a plastic bag (10 × 15 × 0.01 cm) and macerate for 4 min using a rubber mallet.
  CRITICAL STEP: Place the bag on a folded cloth on a flat bench (wooden surface) and press with rubber mallet in a back-and-forth motion. Proper maceration of the rectangular cores guarantees high DNA yield at each of the subsequent step.
4. Add separately 2 mL of either sterile distilled water, boiled sterile distilled water, 1× TE buffer, 1× boiled TE buffer, 1× Tris buffer or 1× boiled Tris buffer to each of the homogenate samples in maceration bags and mix thoroughly for 1 min.
5. Allow the mixture to stand for 10 min at room temperature (23 ± 2 °C).
6. Transfer 1.5 mL of the mixture to 2.0 mL microcentrifuge tube and centrifuge at 11,000× *g* for 90 s at 23 ± 2 °C.
7. Transfer 500 µL of the supernatant to sterile 1.5 mL microcentrifuge tubes and centrifuge at 15,000× *g* for 10 min at 23 ± 2 °C.
8. Discard the supernatants and air-dry the bacterial pellets by inversion of the microcentrifuge tubes on sterile paper towels for 5 min to ensure complete draining off the supernatant.
  CRITICAL STEP: Care should be taken when decanting the liquid solution.
9. Resuspend the bacterial pellets in 1 mL of nuclease-free water, vortex for 30 s and centrifuge at 14,000× *g* at 23 ± 2 °C for 10 min.
10. Discard 800 µL of the supernatants by pipetting and place the remaining 200 µL with the pellets in boiling water bath at 95 °C for 10 min to lyse the bacterial cells.
11. Immediately transfer to −20 °C or cool rapidly on ice.
12. Calibrate the Nanodrop 2000C spectrophotometer (Thermo Fisher Scientific, Wilmington, DE, USA) with nuclease-free water.
13. Measure 1 µL of using a Nanodrop 2000C spectrophotometer on ssDNA-33 mode.
14. Record the concentration in nanograms (ng) and purity (A_260/280_ and A_260/230_) for each sample.
15. Assess the integrity of DNA by running 10 µL of the DNA solution on 0.8% (*w*/*v*) agarose gel dissolved in 1× TAE buffer stained with ethidium bromide solution (1 µg/mL).
16. Immediately use DNA for downstream molecular applications.
17. Store DNA at −20 °C till use
18. Verify the quality and quantity of DNA at three months after extraction.

## 4. Expected Results

### 4.1. Quantitative and Qualitative Assessment of DNA

**Figure 2:**
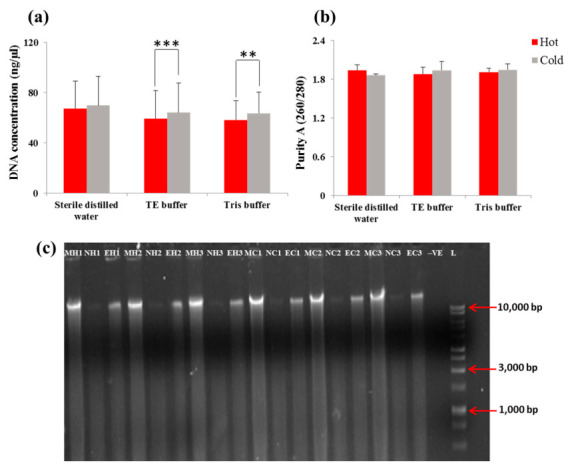
The quantity and quality of extracted genomic DNA based on NanoDrop 2000C spectrophotometer readings and agarose gel electrophoresis. (**a**) Concentration (ng/µL), (**b**) purity of DNA samples expressed as the ratio of absorbance 260/280; three independent biological replicates were used for each of the samples for each of solution/reagent used for DNA extraction, statistical significance, defined as *p* ≤ 0.05; and (**c**) integrity of genomic DNA on 0.8% agarose gel electrophoresis. MH1, MH2 and MH3 = genomic DNA of samples from Meru and DNA isolated using boiled sterile distilled water, TE buffer and Tris buffer, respectively; NH1, NH2 and NH3 = genomic DNA of samples from Nyandarua and DNA isolated using boiled sterile distilled water, TE buffer and Tris buffer, respectively; EH1, EH2 and EH3 = genomic DNA of samples from Uasin Gishu and DNA isolated using boiled sterile distilled water, TE buffer and Tris buffer, respectively; MC1, MC2 and MC3 = genomic DNA of samples from Meru and DNA isolated using cold sterile distilled water at 23 ± 2 °C, TE buffer and Tris buffer, respectively; NC1, NC2 and NC3 = genomic DNA of samples from Nyandarua and DNA isolated using cold sterile distilled water, TE buffer and Tris buffer, respectively, at room temperature (23 ± 2 °C); EC1 and EC2 = genomic DNA of samples from Uasin Gishu and DNA isolated using cold sterile distilled water and TE buffer, respectively, at room temperature (23 ± 2 °C); EC3 = genomic DNA of sample from Uasin Gishu and DNA isolated using previously described method [6]; and L = 1 kb DNA molecular weight marker.

The concentration and purity of DNA were analyzed using a NanoDrop 2000C spectrophotometer. The concentration of obtained genomic DNA from potato tubers infected with *R. solanacearum* using three solutions (water, TE buffer and Tris buffer) for both boiled (hot) and room temperature (cold) conditions, ranged from an average of 58.13 ± 15.37 to 69.93 ± 23.18 ng/µL. Good quality DNA was extracted by the solutions as indicated by the A_260_/A_280_ ratio, which ranged from 1.86 to 1.95. The quality of DNA did not vary significantly for sterile distilled water, TE buffer and Tris buffer between boiled and cold conditions (Figure 2). The highest average DNA concentration of 69.93 ± 23.18 ng/µL was achieved with cold sterile distilled water, with a purity ratio (A_260_/A_280_) of 1.86. There was non-significant difference in DNA concentration and purity between boiled and cold conditions (Figure 2a,b).

The integrity of the isolated DNA was analyzed by agarose gel electrophoresis (Figure 2c). Agarose gels demonstrated intact high molecular weight DNA was extracted by the three solutions for both boiled and cold solutions.

In this study, the concentration and purity of DNA extracts from fresh samples compared with extracts stored at −20 °C for approximately three months. The quality and purity of DNA was presented as A_260/280_ ratio, which ranged from 1.86 to 1.95 (Supplementary Table S1). No significant differences were recorded for values between DNA extracted from fresh potato tubers immediately after sampling and stored DNA samples. Similarly, there were no significant differences between the concentration of the DNA extracted from fresh potato tubers immediately after sampling and the DNA samples that were stored. The quality of the DNA after three months −20 °C was maintained. Both the quantity and quality of DNA in all samples were sufficient for successful PCR amplification with 759/760 primer pair targeting 16S rRNA of bacteria.

### 4.2. Suitability of DNA for PCR Detection of Ralstonia solanacearum

**Figure 3:**
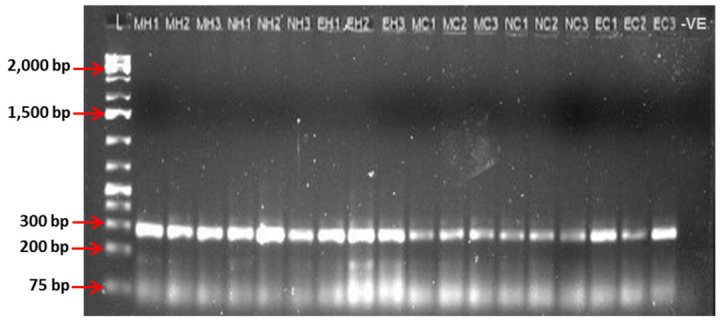
Representative agarose gel (1.5%) showing the amplification of ~280 bp fragment based on 759/760 primer pair targeting 16S rRNA of bacteria. Each lane represents PCR-amplified products from DNA extracted from RSSC-infected potato tubers. MH1, MH2 and MH3 = amplified products of samples from Meru and DNA isolated using boiled sterile distilled water, TE buffer and Tris buffer, respectively; NH1, NH2 and NH3 = amplified products of samples from Nyandarua and DNA isolated using boiled sterile distilled water, TE buffer and Tris buffer, respectively; EH1, EH2 and EH3 = amplified products of samples from Uasin Gishu and DNA isolated using boiled sterile distilled water, TE buffer and Tris buffer, respectively; MC1, MC2 and MC3 = amplified products of samples from Meru and DNA isolated using cold sterile distilled water, TE buffer and Tris buffer, respectively; NC1, NC2 and NC3 = amplified products of samples from Nyandarua and DNA isolated using cold sterile distilled water, TE buffer and Tris buffer, respectively, at room temperature (23 ± 2 °C); EC1 and EC2 = amplified products of samples from Uasin Gishu and DNA isolated using cold sterile distilled water, TE buffer and Tris buffer, respectively, at room temperature (23 ± 2 °C); EC3 was amplified products from genomic DNA extracted using from pure cultures of *R. solanacearum* and used as a positive control; -VE = no amplification for genomic DNA isolated from potato tuber of healthy plants; and L = 1 kb DNA molecular weight marker.

The extracted DNA from the three solutions for both hot and cold (room temperature at 23 ± 2 °C) conditions were used as templates to confirm their corresponding quantity and quality as well as their suitability and applicability for molecular diagnostic assays for detection of *R. solanacearum* in potato tubers. The suitability of DNA solutions for detection and identification of *R. solanacearum* in the samples was performed based on PCR amplification of species-complex-specific fragment of 280 bp. The results produced from PCR amplifications demonstrated that DNA extracted using boiled and cold 1× Tris buffer, 1× TE buffer and sterile distilled water produced the expected PCR-amplified products of ~280 bp (Figure 3). The quality of DNA extracted using the three solutions can be directly amplified by PCR.

### 4.3. Identification of Phylotypes

**Figure 4:**
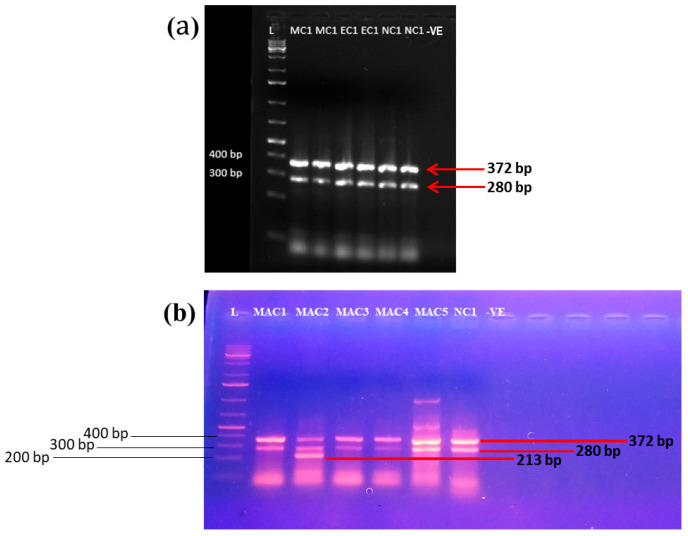
Agarose gel showing the identity and phylotype-specific multiplex PCR amplicons of *R. solanacearum* using DNA extracted by cold sterile distilled water from (**a**) symptomatic and (**b**) asymptomatic potato tuber samples. The *R. solanacearum* isolates generated 280 bp and 372 bp amplicons for *R. solanacearum* identity and phylotype II, respectively. One *R. solanacearum* isolate (MAC2) from asymptomatic sample generated 280 bp, 372 bp and 213 bp amplicons for *R. solanacearum* identity, phylotype II and phylotype IV, respectively. NC1 was the positive control; -VE = no amplification for genomic DNA isolated from potato tuber of healthy plants; and L = 1 kb DNA molecular weight marker.

Since the quality and quantity of DNA did not vary considerably among sterile distilled water, TE buffer and Tris buffer between the boiled and cold extraction solutions, cold water treatment was selected for the identification of the phylotype. Water was selected for extraction of DNA for phylotype identification for both symptomatic and asymptomatic samples because it is readily available and cheaper than TE and Tris buffers. Using multiplex PCR, the expected 372 bp fragments specific to phylotype II *R. solanacearum* were produced for all the DNA samples extracted from *R. solanacearum*-infected potato tubers for both symptomatic and asymptomatic samples (Figure 4).

### 4.4. Sequevar Identification and Phylogenetic Analysis

**Figure 5:**
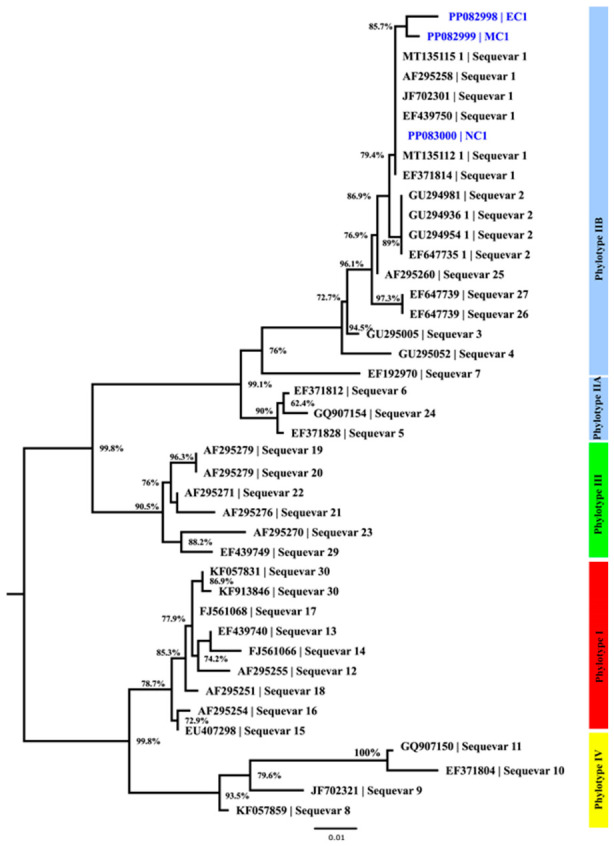
Phylogenetic neighbor-joining tree of *Ralstonia solanacearum* partial *endoglucanase* (*egl*) sequences from the current study (accession numbers in blue color) with reference sequences from the NCBI database. The isolates EC1, MC1 and NC1 were obtained from Uasin Gishu, Meru and Nyandarua, respectively, and the three isolates demonstrated close relationship since they all belong to phylotype IIB and sequevar I.

The PCR amplification of partial *endoglucanase* (*egl*) gene produced the expected fragment of 750 bp. The sequencing chromatograms were of high quality with minimal background noise. In addition, the chromatograms had clean base calling with sharp peaks. Phylogenetic inference of the partial *egl* sequence data placed all the isolates in a clade belonging to phylotype IIB, sequevar 1. The partial *egl* sequences the *R. solanacearum* isolates used in this study clustered with reference sequences of phylotype IIB sequevar 1 strains obtained from the NCBI database and formed a distinct cluster with the phylotype II cluster (Figure 5). Sequences of *R. solanacearum* isolates obtained in the current study were deposited in the NCBI database, with accession numbers PP082998 (for isolate EC1), PP082999 (for isolate MC1) and PP083000 (for isolate NC1).

## 5. Discussion

Bacterial wilt, caused by *R. solanacearum* is a destructive disease of potato, resulting in more than 70% production losses in globally [18]. Therefore, detection of *R. solanacearum* in potato seeds is crucial when aiming to control the dissemination of bacterial wilt. Laboratory testing for certification of seeds before planting is important to confirm the absence of the phytopathogens in planting materials. For the testing to be efficient, there is a need for a robust molecular diagnostic system within developing and even developed economies to aid in the early detection of bulk samples and certification of potato seeds. However, there is no report of a simple and rapid protocol for DNA extraction directly from *R. solanacearum*-infected potato tubers suitable for molecular diagnosis of the pathogen from potato tubers without culture of bacteria and without the need for toxic and hazardous reagents and chemicals. We aimed to develop and demonstrate a simplified and rapid protocol for extraction of DNA from potato tubers infected with bacterial wilt for *R. solanacearum* genetic studies. Genomic DNA of *R. solanacearum* is required for many studies such as molecular detection and identification during seed certification and exchange of seed tubers, exploring genetic diversity and population structures. Genomic DNA in good quantity and quality is the foundation for these studies. With a simple and rapid DNA proposed in this study, conducting such research become feasible in low laboratory settings using basic equipment.

In this study, a simple, quick and rapid DNA extraction from potato tubers infected with *R. solanacearum* has been developed, which is suitable for routine PCR and other molecular diagnostic applications. We prioritized simplicity as well as quality of the extracted genomic DNA for downstream molecular applications like PCR amplification and sequencing, without the need for expensive and toxic chemicals (such as phenol, β-mercaptoethanol, and chloroform) for DNA extraction. Conventional DNA isolation techniques are based on three main principles including disruption of the cytoplasmic and nucleic membranes, purification of DNA and concentration of the DNA [20]. A key step in extraction of genomic DNA is the disruption of the cytoplasmic and nucleic membranes and this can be achieved either by mechanical cell disruption [29] or through rapid boiling of the cell extracts [30]. In this study, mechanical maceration was adequate since DNA was obtained for both boiled and cold solutions. Agarose gel electrophoresis confirmed DNA integrity with strong and well defined bands.

In the current study, genomic DNA of high purity was successfully isolated using water, TE buffer and Tris buffer (average OD260:OD280 ratio ranging from 1.86 to 1.95). In addition, both hot and cold solutions produced intact high molecular weight genomic DNA without shearing of bands for distilled water, TE buffer and Tris buffer. The genomic DNA was of high quality and suitable for downstream molecular applications such as PCR and sequencing. A single amplified band of the expected 280 bp amplicons was obtained confirming the presence of *R. solanacearum* and this finding demonstrated the suitability and applicability of the extracted DNA for PCR-based detection of *R. solanacearum* [14,31]. Furthermore, the successful amplification of the 372 bp amplicons following multiplex PCR placed all the isolates within phylotype II as described in the classification of *R. solanacearum* [32]. The results confirmed that the purity of the genomic DNA was sufficient for molecular diagnostic application for detection of the pathogen and identification of the phylotypes. The *egl* gene is known to code for an important virulent factor and has been used to discriminate members of the RSSC into sequevars [33]. For further elucidation of the taxonomy of the *R. solanacearum*, the DNA isolated using distilled water was used for amplification of the partial *egl* gene and the amplicons successfully sequenced to elucidate the taxonomy of the pathogen. Phylogenetic analysis based on partial *egl* gene sequences confirmed that all the isolates were successfully identified as members of phylotype II sequevar 1 because of their close phylogenetic relationship with reference sequences from the Genbank database supported by high bootstrap values [33]. These findings demonstrate the downstream applicability of the isolated DNA using the developed method.

The storage of conditions of DNA significantly impact on PCR performance, mainly through the degradation of DNA and accumulation of contaminants that can inhibit the polymerase enzyme. In this study, the concentration and purity of DNA extracts from fresh potato tubers immediately after sampling were compared to DNA extracts stored at −20 °C for approximately three months. With an average A_260/280_ ratio in the range from 1.85 to 1.93, the quality of all DNA samples was at an acceptable level with values between 1.8 and 2.0 [34]. Our findings demonstrate the importance of low temperatures at −20 °C for the long-term storage of isolated DNA to ensure both quality and usability in downstream applications including but not limited to PCR and sequencing. Our results indicate that adequate storage of extracted DNA did not have a negative effect on DNA yield in terms of quality, quantity, and integrity, for three months of storage at −20 °C. The DNA samples extracted from fresh potato tubers immediately after sampling as well as the stored DNA extracts, exhibited not only a sufficient DNA amount, but also ensured their purity for PCR applications.

The findings from this study validate the developed DNA extraction protocol especially for the rapid molecular detection of *R. solanacearum*, which has been classified in many countries as a quarantine pathogen [35]. The time used by the current protocol to extract DNA of sufficient quality from tubers was approximately 60 min, which is significantly less compared to the available methods PCR [8,36,37]. Compared with the previous protocols for extraction of genomic DNA which use toxic chemicals such as β-mercaptoethanol, phenol and chloroform [38,39], the current simplified protocol is safe because it does not use toxic chemicals, and hence, the fume hood chamber is not required.

Our findings demonstrate that simple distilled water extraction can obtain sufficient quality of bacterial DNA from plant matrices for molecular diagnosis applications such as PCR and sequencing. Distilled water has an ionic charge that allows it to interact with the DNA, causing the release of the genetic material from the bacterial cells and plant matrix. The centrifugation and washing steps in a simple water extraction dilutes PCR inhibitors such as polysaccharides [40]. It corresponds to an ultra-hypotonic solution, with lower ion concentration, and therefore the interaction between distilled water and DNA is stronger, and extraction from bacteria cells and/or plant matrices is effective. Simple distilled water extraction is effective because it leverages on the high density of bacterial targets and uses physical lysis to remove the inhibitors, making it a reliable, low-cost method for obtaining PCR-compatible bacterial DNA. Asymptomatic potato tubers where disease symptoms were not yet evident and bacterial load that was generally low were used for extraction of DNA using sterile distilled water and confirmed the performance of the protocol in low titer or latent infection. The developed DNA extraction protocol could be used for quarantine purposes and can be adopted by certification programs to ensure distribution of clean potato seeds to farmers.

The DNA extraction procedure described here represents a simple, rapid and inexpensive alternative without use of toxic chemicals compared to conventional DNA extraction protocols and commercial kits for molecular diagnosis of *R. solanacearum*. Several conventional DNA extraction methods (comprising phenol/chloroform extraction, chemical lysis methods with chemical modifications such as CTAB, lithium chloride, SDS, and physical modifications, e.g., bead beating, liquid nitrogen) and commercial kits (spin columns or automated magnetic bead-based procedures) based on various principles have been described and are available for use in routine diagnostics of *R. solanacearum* [41,42]. All of these methods are time consuming (they need several steps for DNA purification, not easily scaled-up for large numbers of samples, or require the acquisition of expensive commercial kits or other equipment) [42,43]. There are some of these kits that are not readily available in Kenya, contributing to an increase in cost due to import tariffs, shipping fees, and time, which takes approximately 8–12 weeks. A disadvantage of methods based on conventional procedures is the use of hazardous chemicals such as phenol/ chloroform. Methods using spin column purification replace these chemicals by guanidine salts; however, these are also hazardous chemicals. In addition, time constraints may make conventional phenol–chloroform extraction of DNA from *R. solanacearum* impractical as additional clean-up procedures may be required to remove carry-over phenols, which inhibit PCRs [44].

The conventional DNA extraction methods and commercial kits are often based on the isolation of *R. solanacearum* from plant material by culturing on semi-selective media, followed by colony identification, morphological and biochemical characterization, and pathogenicity tests. Such tests require one to several weeks before a final confirmation is possible [45]. The simple and rapid protocol developed in the current study, obviated the need for the costly and time-consuming bacteria culture by extraction of DNA directly from the homogenate of potato tubers. This isolated DNA can then be used as a template for polymerase chain reaction (PCR), thus circumventing the need for culturability [40].

This study has notable limitations. The DNA extraction procedure in the current study was not compared with conventional DNA extraction methods and commercially available kits in terms of DNA extraction efficiency, repeatability, and reproducibility. Therefore, comparison experiments with the standard CTAB method or commercial DNA extraction kits were not conducted in parallel to demonstrate the superiority or equivalence of this method. In addition, the genomic DNA extraction protocol was not validated and therefore needs to be tested and validated/verified on larger number of field samples. Due to the ability of *R. solanacearum* to infect a wide range of hosts, further studies to test the applicability of the method to samples with different hosts should be evaluated.

## 6. Conclusions

The simplified protocol developed in this study is simple, fast and effective for routine extraction of sufficient genomic DNA with high quality from potato tubers infected with *R. solanacearum*. We demonstrated that the extracted genomic DNA extracted using the developed protocol was suitable for downstream molecular diagnostic applications such as PCR, multiplex PCR and DNA sequencing. The DNA isolation protocol could also be implemented in resource-limited settings, especially developing countries. The developed DNA extraction protocol could be used for routine diagnostics and can be adopted by certification programs to ensure distribution of clean potato seeds to farmers. Since cold distilled water produced the highest average concentration of DNA and boiling did not significantly improve the metrics, we recommend the use of cold distilled water method as an accessible and simplest way to obtain DNA for routine diagnostics of *R. solanacearum*. The use of cold distilled water method is the simplest because it does not require a buffer or a microwave. The method is suitable for the rapid diagnosis of *R. solanacearum* in asymptomatic and symptomatic potato tubers.

## Figures and Tables

**Table 1:** Nucleotide sequences of primers used for PCR amplification of species, phylotypes and sequevars of *Ralstonia solanacearum* species complex.

Primer Name	Sequence (5′–3′)	Size (bp)	Specificity
759R	GTCGCCGTCAACTCACTTTCC	282	Species validation
760F	GTCGCCGTCAGCAATGCGGAATCG		
Nmulti:21:1F	CGT TGA TGA GGC GCG CAA TTT	144	Forward primer for identification of phylotype I
Nmulti:21:2F	AAG TTA TGG ACG GTG GAA GTC	372	Forward primer for identification of phylotype II
Nmult:23:AF	ATTACGAGAGCAATCGAAAGATT	91	Forward primer for identification of phylotype III
Nmult:22:InF	ATTGCCAAGACGAGAGAAGTA	213	Forward primer for identification of phylotype IV
Nmult:22:RR	TCG CTT GAC CCT ATA ACG AGT A		Reverse primer for identification of ALL phylotypes (I–IV)
Endo-F Endo-R	ATG CAT GCC GCT GGT CGC CGC GCG TTG CCC GGC ACG AAC ACC	750	*egl* gene for sequevar identification

Adapted from Fegan and Prior [14] and Fegan et al. [23].

## Data Availability

All data generated or analyzed during this study are included in the manuscript. The nucleotide sequences have been deposited in the NCBI database with accession numbers PP082998 (for isolate EC1), PP082999 (for isolate MC1) and PP083000 (for isolate NC1).

## Supplementary Materials

The following supporting information can be downloaded at: https://www.mdpi.com/article/10.3390/mps9030084/s1, Table S1: Concentration, A260/A280 and A260/A230 ratios of DNA extracted using different reagent/solution, measured using the NanoDrop™2000/2000c Spectrophotometer.
