# Supplementary material for: A Simple, Rapid and Reliable Protocol for Extraction of High Quality Bacterial Genomic DNA Directly from Potato Tubers for Efficient PCR-Based Surveillance and Molecular Characterization of Ralstonia solanacearum
Source: Methods Protoc. 2026 May 31;9(3):84. doi: 10.3390/mps9030084 (PMC13304767; doi:10.3390/mps9030084)
Supplement: Supplementary file 1 [file mps-09-00084-s001.zip › mps-4139956-supplementary.pdf]

Supplementary Material

Protocol

A Simple, Rapid and Reliable Protocol for Extraction of High Quality Bacterial Genomic DNA Directly from Potato Tubers for Efficient PCR-Based Surveillance and Molecular Characterization of *Ralstonia solanacearum*

Brian Mwangi [1,2], Joshua Njiru [3,*], Sarah Wandili [2], Kennedy Gachoka [2], Kenneth Mburu [4], Geoffrey Muriira [3], Henry Rotich [3], Elvince Ager [1] and Evans N. Nyaboga [1,*]

[1] Department of Biochemistry, University of Nairobi, Nairobi P.O. Box 30197–00100, Kenya; bmwangi918@gmail.com (B.M.); agerelvince@gmail.com (E.A.)
[2] Department of Biological Sciences, Meru University of Science & Technology, Meru P.O. Box 972–60200, Kenya; swandili@must.ac.ke (S.W.); kgachoka@must.ac.ke (K.G.)
[3] Research and Development, Kenya Bureau of Standards, Nairobi P.O. Box 54974–00200, Kenya; muriirag@kebs.org (G.M.); rotichh@kebs.org (H.R.)
[4] Department of Life Sciences, South Eastern Kenya University, Kitui P.O. Box 170–90200, Kenya; kmburu@seku.ac.ke
* Correspondence: njiruj@kebs.org (J.N.); nyaboga@uonbi.ac.ke (E.N.N.)

List of Supplementary Tables

Table S1. Concentration, A260/A280 and A260/A230 ratios of DNA extracted using different reagent/solution, measured using the NanoDrop™2000/2000c Spectrophotometer (Thermo Scientific™).

		Measured immediately after extraction			Measured after storage of DNA extracts at -20 °C for three months		
Reagent used for DNA extraction	Sample	Concentration (ng/µl)	A260/280	A260/230	Concentration (ng/µl)	A260/280	A260/230
Hot water	MH1	90.5±1.22	1.92±0.12	2.12±0.15	89.2±1.00	1.94±0.33	2.03±0.02
	NH1	47.4±2.41	2.03±0.41	2.10±0.21	48.4±2.66	1.98±0.21	2.31±0.11
	EC1	64.3±1.17	1.87±0.17	1.97±0.17	64.9±1.11	1.89±0.11	2.07±0.22
Cold water	MH1	94.6±2.51	1.88±0.51	2.18±0.33	94.1±2.91	1.91±0.02	2.23±0.44
	NH1	48.6±1.44	1.84±0.44	2.14±0.34	48.8±1.11	1.88±0.40	2.11±0.39
	EC1	66.6±3.22	1.87±0.22	2.20±0.00	65.9±3.43	1.81±0.05	2.22±0.04
	MAC1	83.3±2.44	1.93±0.21	2.13±0.21	ND	ND	ND
	MAC2	88.6±1.51	1.94±0.53	2.20±0.14	ND	ND	ND
	MAC3	96.1±2.11	1.91±0.12	2.12±0.13	ND	ND	ND
	MAC4	91.7±1.76	1.87±0.32	2.11±0.11	ND	ND	ND
	MAC5	68.9±0.99	1.89±0.33	2.19±0.23	ND	ND	ND
Hot TE	MH2	84.2±4.21	1.99±0.82	2.09±0.44	83.4±3.44	1.93±0.22	2.19±0.41
	NH2	41.4±2.32	1.79±0.45	1.99±0.40	41.9±3.01	1.89±0.10	2.21±0.43
	EC1	52.8±1.44	1.87±0.67	2.37±0.11	52.1±1.99	1.81±0.07	2.44±0.11
Cold TE	MH2	80.8±4.32	2.05±0.88	2.13±0.11	79.6±3.25	2.11±0.33	2.19±0.14
	NH2	37.2±1.24	1.98±0.73	1.98±0.31	37.0±1.44	1.88±0.41	1.89±0.36
	EC1	74.9±3.11	1.79±0.54	2.37±0.42	74.1±3.71	1.87±0.22	2.11±0.22
Hot Tris	MH3	74.9±2.51	1.84±0.72	2.44±0.82	74.3±2.01	1.92±0.51	2.21±0.02
	NH3	44.7±1.52	1.92±0.51	2.02±0.46	44.9±1.00	1.91±0.22	2.45±0.16
	EC4	54.8±2.32	1.97±0.44	1.98±0.41	53.3±2.11	1.91±0.34	2.08±0.49
Cold Tris	MH3	81.3±3.13	1.99±0.33	2.19±0.43	80.8±3.81	1.88±0.36	2.14±0.33
	NH3	47.9±0.52	2.01±0.45	2.21±0.55	47.1±1.12	1.91±0.21	2.29±0.15
	EC4	61.9±3.12	1.85±0.37	2.05±0.67	62.5±2.08	1.95±0.09	2.26±0.34
Positive control DNA sample extracted from bacteria	EC3	148.2±3.49	1.93±0.04	2.25±0.02	147.4±4.22	1.92±0.41	2.18±0.17

MAC1 – MAC5 are samples collected from asymptomatic potato tubers and plants. ND = Not done
